# Feasibility of Valorization of Post-Consumer Recycled Flexible Polypropylene by Adding Fumed Nanosilica for Its Potential Use in Food Packaging toward Sustainability

**DOI:** 10.3390/polym15051081

**Published:** 2023-02-21

**Authors:** Eliezer Velásquez, Carol López de Dicastillo, Cristian Patiño Vidal, Guillermo Copello, Cristopher Reyes, Abel Guarda, María José Galotto

**Affiliations:** 1 Packaging Innovation Center (LABEN-Chile), University of Santiago of Chile (USACH), Santiago 9170201, Chile; 2 Center for the Development of Nanoscience and Nanotechnology (CEDENNA), University of Santiago of Chile, Santiago 9170124, Chile; 3 Packaging Laboratory, Institute of Agrochemistry and Food Technology (IATA-CSIC), 46980 Paterna, Spain; 4 Universidad de Buenos Aires, Facultad de Farmacia y Bioquímica, Departamento de Ciencias Químicas, Junín 956, Buenos Aires C1113AAD, Argentina; 5 CONICET—Universidad de Buenos Aires, Instituto de Química y Metabolismo del Fármaco (IQUIMEFA), Junín 956, Buenos Aires C1113AAD, Argentina; 6 Food Science and Technology Department, Technological Faculty, University of Santiago of Chile (USACH), Santiago 9170201, Chile

**Keywords:** post-consumer polypropylene, fumed silica, food packaging, recycling

## Abstract

The food industry has a current challenge of increasing the recycling of post-consumer plastics to reduce plastic waste towards a circular economy, especially flexible polypropylene, which is highly demanded in food packaging. However, recycling post-consumer plastics is limited because service life and reprocessing degrade their physical-mechanical properties and modify the migration of components from the recycled material to the food. This research evaluated the feasibility of valorization of post-consumer recycled flexible polypropylene (PCPP) by incorporating fumed nanosilica (NS). For this purpose, the effect of concentration and type (hydrophilic and hydrophobic) of NS on the morphological, mechanical, sealing, barrier and overall migration properties of PCPP films was studied. Incorporating NS improved Young’s modulus and, more significantly, tensile strength at 0.5 wt% and 1 wt%, where a better particle dispersion was confirmed by EDS-SEM, but it diminished elongation at breakage of the films. Interestingly, NS tended to increase the seal strength of PCPP nanocomposite films more significantly at higher NS content, showing a seal failure of the adhesive peel type which is preferred for flexible packaging. NS at 1 wt% did not affect the water vapor and oxygen permeabilities of the films. Overall migration of PCPP and nanocomposites exceeded the limit value of 10 mg dm^−2^ allowed by European legislation at the studied concentrations of 1% and 4 wt%. Nonetheless, NS reduced the overall migration of PCPP from 17.3 to 15 mg dm^−2^ in all nanocomposites. In conclusion, PCPP with 1 wt% of hydrophobic NS presented an improved overall performance of the studied packaging properties.

## 1. Introduction

Packaging is a key technology for the conservation of food properties since it covers the handling, distribution, storage and consumption of food. Each food requires packaging with specific physical properties depending on its characteristics and deterioration processes. Although plastic is the material that has responded well to these requirements, it presents two concerns: a) mass transfer processes, highlighting the permeability of gases and/or water vapor and migration processes of compounds from the packaging to the food, and b) the inadequate management of the plastic waste that is causing serious environmental pollution and the loss of valuable resources [1,2]. Thus, the main challenges in food packaging are limiting mass transfer processes and changing the nature of packaging production towards sustainable materials. 

Using recycled plastics is a mandatory path towards sustainability in the coming years, overcoming the technological challenges that this entails. Mechanical recycling is the most convenient method from an operational, economic and environmental point of view [3,4]. However, thermal-mechanical oxidation during reprocessing produces structural changes depending on the type of polymer, the conditions and the reprocessing cycles. Chain scission (polypropylene, PP, and polyethylene terephthalate, PET), crosslinking (polyethylene, PE), formation of recombined structures (polystyrene, PS), esterification and degradation by hydrolysis (polylactic acid, PLA) are some of the main degradation reactions which prevail for each mentioned polymer [5]. They modify the molar mass distribution of the polymers which leads to a deterioration of their physical properties, mainly mechanical and barrier. In addition, low molar mass chains and other substances can affect the chemical safety of the plastic material and increase the migration of components to the food, modifying the barrier properties for packaging applications [6,7]. This fact can limit its application as food packaging because it does not offer adequate protection and affects food quality and safety. Therefore, studying and evaluating the effect of using recycled plastics for packaging materials and seeking solutions to improve the physical properties of post-consumer plastics and ensure their safety for food packaging applications is a current need.

PP is used by the food industry to package foods as nuts, butter, snacks, pasta, and dairy products, among others. Its attractive properties such as low specific weight, low cost, good sealability, moisture barrier and an adequate balance between stiffness and impact resistance, create a high demand for PP and the industry has a great interest in improving its low oxygen barrier properties to expand its usage in packaging materials for oxygen-sensitive foods [8,9]. Nonetheless, as was already mentioned, polyolefins degrade during the packaging’s service life and recycling process due to the thermal and shear stress cycles which they are subjected. The type and source of the polyolefin and processing conditions determine the degradation reaction types and their impact on the physical-mechanical properties [10,11]. In this context, PP reprocessing leads to deterioration in the polymer viscosity and essential parameters for flexible packages, such as Young’s modulus (stiffness), elongation at break (ductility) and tensile strength; properties needed for rigid containers, such as impact resistance, are also weakened [5]. 

Moreover, low molar mass chains and other substances in the recycled polymer can affect its chemical safety, increase the migration of components to the food and modify the barrier properties for packaging applications [6]. Most works have simulated the recycling process by reprocessing virgin PP at a laboratory scale or using post-industrial PP, which comprises a controlled polymeric system without pollutants. Therefore, it is highlighted that research is required to study reprocessing and valorization of post-consumer recycled polypropylene (PCPP), which is a more complex system in terms of composition, chemical structure, and chemical safety due to the presence of pollutants incorporated during service life and recycling, considering that these characteristics depend on the plastic waste source. Even more, attention has recently focused on valorizing post-consumer flexible PP due to the low recycling rates of this material compared to rigid PP which is easier to collect and reprocess. 

Developing nanocomposites from recycled polymers to improve their physical-mechanical and barrier properties by incorporating nanoparticles at low concentrations in the plastic matrix has been a current attractive alternative [12]. The improvement in the performance of the polymer matrix is due to a high polymer-nanoparticle interfacial area. Thus, nanoclays, calcium carbonate and carbon nanotubes have been incorporated into PP to study the effect on the thermal-mechanical properties [13]. Increments of the tensile strength and elastic modulus have been reported by nanoclay addition up to 3 wt% but the tendency changed at higher concentrations due to changes in the clay dispersion [14]. Meanwhile, incorporating multi-wall carbon nanotube in PP up to 1.5 wt% increased the impact resistance [15]. The nanocomposite’s resulting properties will depend on the polymer type, preparation method, concentration and dispersion of the nanoparticles, among others [12,16,17]. High costs and safety issues are the main limitations of using some nanoparticles in food packaging [18]. In this context, fumed silica nanoparticles (NS) result in an outstanding alternative thanks to their commercial availability, low cost and use as a food additive [19].

NS exist in a wide range of sizes (specific area ranging from 50 to 400 m^2^/g) and with various surface treatments from hydrophilic to hydrophobic. Due to their fractal structure and the high specific area, fumed silica is subject to self-aggregation and can consequently form a network of connected particles in the molten polymer [20,21], promoting significant changes in physical properties such as as rheological parameters. Amorphous synthetic silica has recently been authorized as a food additive by the European Union under the code E551 [22,23] and the Food and Drug Administration [19]. Meanwhile, its use as a filler of polymers for food packaging applications is allowed in its bulk form, silanated or not [24,25]. Recent publications indicated that nano-SiO_2_ potentially increased the barrier properties of virgin poly(3-hydroxybutyrate-*co*-3-hydroxyhexanoate) (PHBH) obtained by casting and delayed microbial growth in shrimps and loquat fruit when silica was incorporated in virgin low-density polyethylene (LDPE) [26,27,28]. Nonetheless, few studies on polyolefin-based nanocomposites using fumed nanosilica have been developed for food packaging applications [29]. PP/NS nanocomposites have been only obtained using virgin PP by hydraulic pressing, compression molding or fiber drawing processes, which are not the typical industrial methods for food packaging manufacturing, reporting only thermal and rheological properties with variable trends according to the polypropylene source, NS type and concentration and processing conditions [30,31,32]. According to our review, nanocomposites of post-consumer recycled flexible polypropylene and fumed silica have not been published. Therefore, research on the potential role of NS in the valorization of PCPP for food packaging applications is required to advance a potentially sustainable alternative to increase the recycling rates of this post-consumer plastic.

In this work, nanocomposite films based on PCPP and NS were developed through extrusion in order to evaluate their physical-mechanical performance, barrier and migration properties for food packaging. For this purpose, the effect of two types of fumed silica (hydrophilic and hydrophobic) and their concentration in the PCPP performance were studied. Morphological, mechanical and barrier properties (water vapor and oxygen permeability) were analyzed. Sealing conditions, seal strength, seal failure type, and overall migration studies of the developed nanocomposite films were also carried out in order to verify the viability of using these nanocomposites in food packaging.

## 2. Materials and Methods

### 2.1. Materials

Homopolymer grade virgin polypropylene in pellets (VPP) (MFI = 3.0 g 10 min^−1^ at 230 °C and 2.16 kg) was supplied by Petroquim S.A. (Santiago, Chile). Post-consumer recycled polypropylene pellets (PCPP) obtained from flexible PP were purchased from Inproplas S.A (Santiago, Chile). Two types of commercial fumed nanosilica (NS) purchased from Haochuang Material (Shandong, China), with native particle size ranging from 5 nm to 40 nm, were used: (i) hydrophilic nanosilica (NS1) with a specific surface area of 200 m^2^ g^−1^, and (ii) hydrophobic nanosilica (NS2) produced by NS1 after chemical post-treatment with dimethyldichlorosilane.

### 2.2. Preparation of PCPP/NS Nanocomposite Films

Nanocomposite films based on PCPP and fumed nanosilica were obtained through extrusion. NS concentrations of 0.5, 1, 2 and 4 wt% with respect to the total nanocomposite mass were used, and films were designed as PCPP-XNSY where X is the weight concentration of the corresponding fumed nanosilica NSY, hydrophilic (NS1) or hydrophobic (NS2). Control PCPP and VPP films were also obtained. VPP was used as a commercial reference of a type of polypropylene used in the manufacture of bioriented extruded or coextruded films. The films were produced in a twin-screw extruder Labtech Scientific LTE-20–40 (Samutprakarn, Thailand) with a temperature profile from 180 °C to 195 °C from feeding to the extruder die. The screw speed was 35 rpm and the torque was between 40% to 50%. Before extrusion, the polymer and the NS were dried at 100 °C for 24 h, and then, pre-mixed manually through stirring until homogenization. The thickness of the films was measured by using of digital micrometer Digimatic Mitutoyo ID-C112 (Kawasaki, Japan), resulting between 150 and 180 µm. 

### 2.3. Characterization of the Nanocomposite Films

#### 2.3.1. Scanning Electron Microscopy (SEM) and Energy Dispersive Spectroscopy (EDS)

The distribution of NS in the nanocomposite films was assessed by means of EDS elemental mapping. Samples were coated with graphite before analyzing using a SEM (FEI Quanta-200, FEI, Hillsboro, OR, USA) equipped with an EDS analyzer (Oxford Instruments). Particle sizes and lengths in the images were measured using ImageJ open source software (https://imagej.nih.gov/ij/, access date: December 2022). 

#### 2.3.2. Tensile Test 

The effect of NS incorporation on the Young’s Modulus (YM), tensile strength (TS) and elongation at break (EB) of the PCPP was evaluated through a universal machine Zwick Roell Proline BDO-FB 0.5 TH (Ulm, Germany) following the ASTM D882 standard normative. Ten specimens of each film (16 cm × 2.5 cm) were cut and conditioned at 23 °C and 50% HR for 48 h. A load cell of 500 N, a separation distance between the jaws of 50 mm and a crosshead speed of 500 mm min^−1^ were used for the test. 

#### 2.3.3. Seal Strength 

The seal strength of PCPP and the nanocomposites was evaluated as follows: 

First, the nanocomposites were sealed using a sealing machine Labthink HST-H3 (Medford, OR, USA) following the ASTM F2029 standard. normative The optimum conditions for sealing the nanocomposites (sealing temperature, time and pressure) were found from the seal curve built for the control PCPP film. For this purpose, six specimens (16 cm × 2.5 cm) were sealed in a temperature range between 148 °C to 154 °C, using sealing times of 1.5, 2 and 2.5 s at a constant pressure of 350 kPa. The seal curve of the VPP was also constructed under the same conditions for a rough comparison. Samples were conditioned at 50% RH for 48 h. 

Subsequently, the seal strength of the nanocomposites was determined using a universal machine Zwick Roell Proline BDO-FB 0.5 TH (Ulm, Germany), following the ASTM F88/F88 M-21 standard. The test conditions were: a load cell of 500 N, a separation distance between the jaws of 25 mm and a crosshead speed of 250 mm min^−1^ using the non-supported seal technique. The seal strength was determined as the average of six measurements calculated in the data range of 80% central values of the sealing profile plot (force vs. displacement) using the program TestXpert III

#### 2.3.4. Water Vapor Permeability (WVP) 

WVP of the control PCPP and the nanocomposite films which resulted in the best mechanical performance (1 wt% of NS) were analyzed in duplicate. Water vapor transmission rate (WTR) of the films with a contact area of 5.5 cm^2^ was determined at 37.8 °C and 90% RH with an analyzer Permatran Mocon W 3/34 (Minneapolis, MN, USA) according to the F1249-20 standard. Films were put into permeability cells, and the WVTR values were plotted until they reached a constant value. The WVP was calculated using Equation (1):

(1) $$\mathrm{WVP}=\mathrm{WVTR}*L/P_{W}$$

where WVTR is the water vapor transmission rate (g m^−2^ s^−1^), L is the thickness of the films (mm) and P_W_ is the gradient of partial pressure of water vapor (Pa).

#### 2.3.5. Oxygen Permeability (OP)

The oxygen transmission rate (OTR) of the control PCPP and the nanocomposite films were determined in duplicate at 23 °C and 0% RH with an analyzer Oxtran Mocon ST 2/21 (Neuwied, Germany) according to the ASTM D-398517 standard. Films supported in permeability masks were prepared and put into the permeability cells. OTR values were determined for 24 h. Previously, the samples were purged with nitrogen to be posteriorly exposed to an oxygen flow rate of 10 mL min^−1^. The OP was calculated using Equation (2):

(2) $$\mathrm{OP}=\mathrm{OTR}*L/P_{O2}$$

where OTR is the oxygen transmission rate (cm^3^ m^−2^ day^−1^), L is the thickness of the films (mm) and P_O2_ is the partial pressure of oxygen (atm).

#### 2.3.6. Overall Migration (OM)

Overall migration tests in a fatty simulant were carried out on the control PCPP and nanocomposite films with NS at 1% and 4 wt% according to EU Regulation N° 10/2011 and Chilean standard NCH 3367/2. Olive oil was used as a food simulant since the great affinity between PP and the fat simulant would promote a maximum overall migration of substances from the developed films. Glass tubes were filled with extra virgin olive oil “Chef” (maximum acidity 0.3%) as fatty food simulant. The total immersion of the films into the oil was done with an area/volume ratio of 6 dm^2^ L^−1^. The films were fixed in metallic supports to avoid direct contact with the tube walls. The food simulant and the samples were maintained in contact for ten days at 40 °C. Subsequently, the films were withdrawn and cleaned to remove the excess oil and subjected to an extraction process of the food simulant by Soxhlet extraction. After that, the extracted oil was subjected to a methylation process, and it was quantified with a gas chromatography PerkinElmer Gases Clarus 500 equipped with a flame ionization detector (FID) and a capillarity column Elite-Wax (30 m × 0.32 mm ID × 0.25 µm film thickness). The volume injection was 1 µL and a split of 50 mL min^−1^. The oven temperature started at 160 °C for 5 min, and then it was raised to 220 °C at 15 °C min^−1^. After 4 min, the temperature was increased at 20 °C min^−1^ until 250 °C and maintained for 1 min. Helium was used as a carrier at 10 psi. The injector and detector temperatures were fixed at 250 °C and 260 °C, respectively. Fatty acids such as: palmitic (C16), palmitoleic (C16.1), heptadecanoic (C17), stearic (C18), oleic (C18.1) and linoleic (C18.2) were detected and quantified. In this analysis, the fatty acids determined from the samples indirectly allowed calculation of the amount of plastic that migrated to the fatty simulant through the difference between the initial and final weights of the films. The analysis was carried out by duplicate. The fatty acids were quantified using a calibration curve previously prepared from five methylated solutions of extra virgin olive oil with weights between 0.0055 and 0.1132 g (R^2^ = 0.99). OM values were reported as the average and standard deviation of two measurements. 

#### 2.3.7. Statistical Analysis

The results obtained for the tensile test, seal strength, WVP, OP and OM were statistically analyzed through a variance analysis (ANOVA) and LSD Fischer’s multiple range test in order to find statistically significant differences between the samples for a random experimental design (*p* < 0.05).

## 3. Results and Discussions

### 3.1. Scanning Electron Microscopy and Energy Dispersive Spectroscopy (EDS)

**Figure 1:**
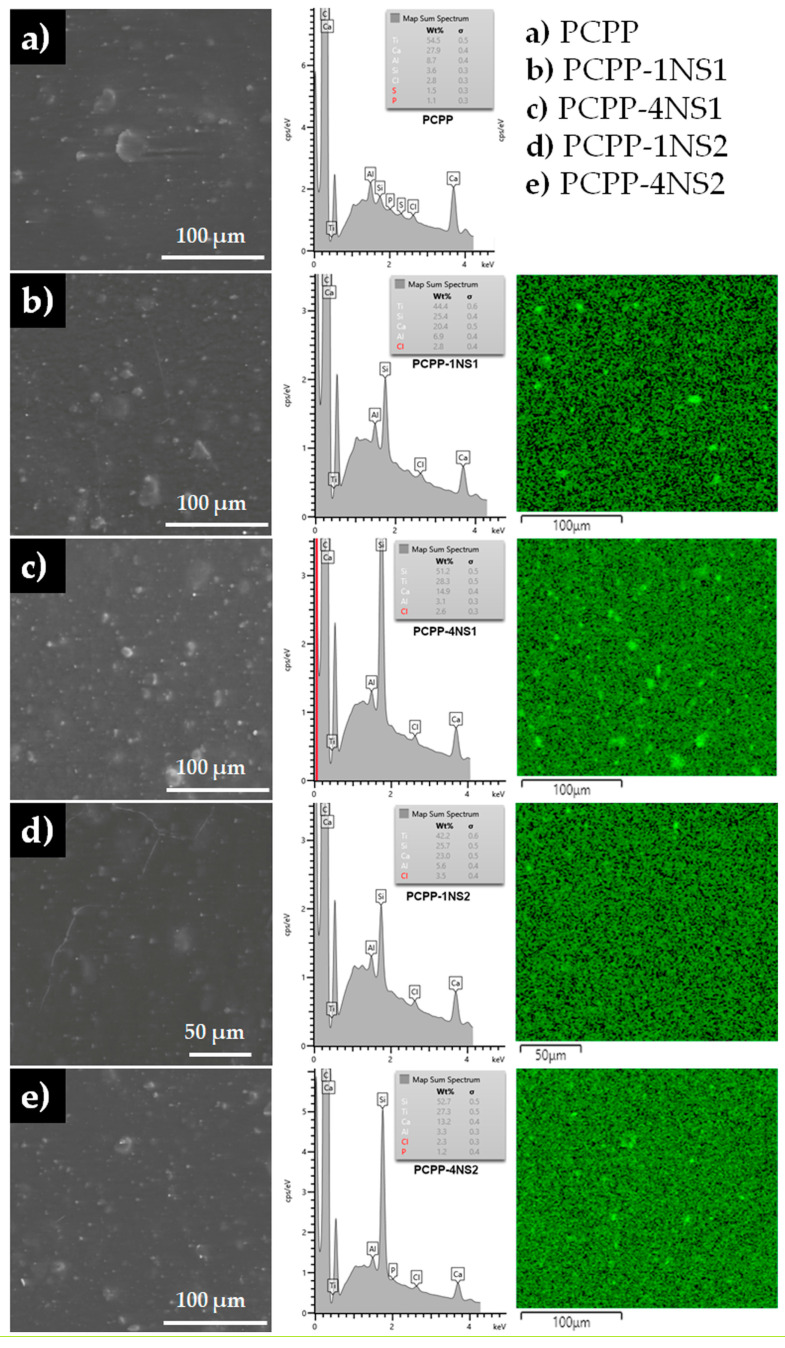
ESEM micrographs and EDS elemental mapping of (**a**) PCPP control and (**b**–**e**) nanocomposite films. Left, middle and right columns show electron images of surface topography, map sum spectra of elements and EDS images for Si, respectively.

SEM images of the control and the nanocomposite films show flat and non-porous surfaces with no noticeable cracks (Figure 1, left column). All images showed topography variations due to the presence of particles/polymer agglomerates with sizes around 1 to 20 µm. These particles appeared on the surface of the control PCPP and in the nanocomposite films, which indicated that their origin is related to pre-existent constituents from the PCPP and not to the formation of silica nanoparticle agglomerates.

In order to confirm the absence of large nanoparticle agglomerates and evaluate their distribution in the films, EDS elemental mapping was performed. Figure 1 (middle and right columns) shows the mapping of elements indicating the location and density of silicon. As it was expected, the PCPP-4NS films showed the highest amount of Si among the samples, followed by the PCPP-1NS films. PCPP films exhibit a minimum amount of Si compared with the nanocomposites. The presence of this mineral in the recycled polymer would be related to the cross-contamination considering the ubiquity of Si in the environment.

Silicon mapping also indicates that the nature of NS particles influenced their distribution in the PCPP matrix. For the lower NS content (PCPP-1NS1), NS1 was mildly agglomerated. This can be observed as higher density zones (brighter spots) in the images. The higher amount of this NS in the PCPP-4NS1 film produced a higher number of these zones with larger sizes. Probably, the higher the NS content, the poorer the distribution in the polymeric material. This finding was not observed for films containing NS2, where a more homogeneous distribution of Si could be observed at both loading levels. With the NS2 surfaces being organically modified, their compatibilization with PCPP moieties would be more efficient, leading to more homogeneous composites and fewer agglomerates formation.

### 3.2. Mechanical Properties

The mechanical parameters of the films are shown in Table 1. Young’s modulus (YM) is related to the stiffness of the material, tensile strength (TS) is the maximum force that the material supports before being broken, and the elongation at break (EB) is the ability of the material to support deformation without breaking [33]. 

The YM and TS of PCPP were lower than VPP due to: (i) the presence of polyethylene (PE) and traces of other contaminants, as it was verified with ATR-FTIR and DSC analyses (see Figures S1 and S2, Supplementary Materials), which could affect the elasticity modulus and generate stress points since they would not be completely incorporated in the polymeric matrix, and (ii) PCPP did not come from exclusive recycling of the commercial VPP used as reference but was a blend of post-consumer PP from different flexible packaging. During the recycling of polymers, degradation reactions occur, and in the case of PP, the reduction of its molar mass by the effect of thermo-mechanical cycles predominates. This, in turn, negatively affects the stiffness of the polymeric matrix since the shorter chains generate smaller crystalline zones [16]. This phenomenon was also reported by Raj et al. (2013) in their study about the effect of recycling cycles in virgin PP [34]. The presence of PE could also affect the stress distribution in the film due to differences in miscibility between PE and PP. Furthermore, the low stiffness of PCPP compared to VPP would also be associated with the low crystallinity of the recycled polymer as determined by DSC analysis (Figure S2, Supplementary Materials). Regarding the elongation at breakage point, PCPP showed values slightly higher than VPP but without statistical differences. This effect is associated with the presence in the PCPP of PE which is more ductile [7]. High EB values have been previously reported for LLDPE. For instance, Wu and Wang (2020) reported an EB higher for pure LLDPE (606%) than for pure PP (200%) determined by the ISO37 standard [35]. 

For the nanocomposites, improvements in the YM and the TS of films were obtained at 1 wt% of NS since a better dispersion and interaction of nanofillers with the polymeric matrix and a good interfacial adhesion was obtained, as SEM analysis evidenced (Figure 1). Concentrations of NS higher than 1 wt% decreased the YM and TS due to a possible agglomeration of the nanoparticles, with a statistically significant difference for PCPP-4NS1. Recent research on nanocomposites of PP and NS also reported improvements in the mechanical properties of the polymer but using virgin PP and techniques other than film extrusion. Titone et al. (2021) evidenced an increase of YM and TS of virgin homopolymer PP obtained by compression molding with the incorporation and the increment of concentration up to 2 wt% of a nanosilica modified by hexamethyldisilazane [32]. Similarly, YM and TS of nanocomposites obtained by hot-pressing of homopolymer PP were gradually increased when NS was incorporated and the concentration increased up to 10 wt% [36]. In both studies, the authors associated the increase of the mechanical parameters with the good dispersion of the nanoparticles into the polymeric matrix.

On the other hand, NS addition reduced EB, possibly due to the presence of agglomerates of NS that hindered the dissipation of the energy along the polymeric film favoring a premature break of the PCPP (Table 1) [37]. It has been reported that EB is affected by the incorporation of nanofillers. Recently, it has been reported that EB of virgin PP obtained by compression molding was reduced by 83% approx. through adding 2 wt% of NS modified with hexamethyldisilazane [32]. Meanwhile, in our work, the incorporation of NS modified by dimethyldichlorosilane caused a reduction of EB in the PCPP which was less significant and equal to 40%. An exception was the nanocomposite PCPP-4NS1, which exhibited the lowest EB of 9% since the hydrophilic nature of NS1 possibly caused a higher amount of agglomerates and triggered points of break by a more weak polymer-silica adhesion. 

It is important to highlight that the high hydrophobicity of NS2 and its miscibility with the PCPP favored a better distribution of the energy in the polymeric matrix and a higher energy transference to the nanoparticle [38]. For this reason, NS2 caused a slight tendency to increase the YM and TS of PCPP compared to NS1 at similar concentrations. 

### 3.3. Seal Strength 

#### 3.3.1. Sealing Curves

**Figure 2:**
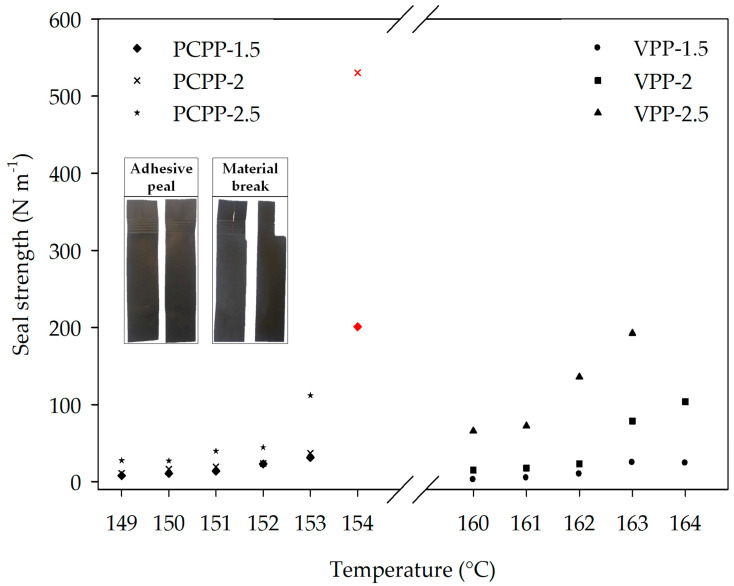
Sealing curves of commercial VPP and control PCPP films, designated as VPP-X and PCPP-X where X is the sealing time. Red symbols correspond to the temperature and time conditions where there was a failure “material break” type.

One of the main functionalities of a flexible film for packaging is the maximum seal strength which is defined as the maximum force per width of the seal required to progressively peel it under some specific test conditions. Figure 2 shows the sealing curves of PCPP and VPP films constructed to obtain the optimum sealing temperature and time for the nanocomposites at 350 kPa. An increase in the sealing time increased the sealing strength of all films. The initial sealing temperatures of PCPP and VPP were 149 °C and 160 °C, respectively. Films exhibited adhesive failures under all conditions according to the ASTM F88/F88M-21 standard, except under some conditions where a material break occurs, as is shown in the insert of Figure 2 (symbols with red color). In the VPP film, the sealing time of 1.5 s did not show substantial changes in the seal strength with increased temperature. However, a sealing time of 2 s considerably increased the seal strength at the highest temperatures. Thus, around a 3-fold increase was obtained in the seal strength of VPP film at 163 and 164 °C. On the other hand, a sealing time of 2.5 s maintained the seal strength of the film unchanged up to 161 °C; subsequently it exponentially increased up to 163 °C, and finally, a distortion of the film occurred at 164 °C. 

Conversely, PCPP required lower temperatures to be sealed compared to VPP (Figure 2). In this context, temperatures between 149 °C and 153 °C allowed the sealing of the PCPP film, and a maximum seal strength of 108 N m^−1^ was determined at 153 °C and 2.5 s. The reduction of the sealing temperature in the PCPP with respect to VPP would be associated with the PP grade and the presence of PE traces that were confirmed by ATR-FTIR and DSC analysis (Figures S1 and S2, Supplementary Materials), considering that the recycled polymer presented melting transitions (105 °C to 130 °C) lower than the VPP [39,40]. Furthermore, a seal strength greater than the strength that supports the material during the stress test at 154 °C was observed for all sealing times, which promoted the film’s distortion and a material break failure (red symbols in Figure 2). According to these results, 153 °C and 2.5 s were selected as the better sealing conditions to evaluate the seal strength of the nanocomposites.

#### 3.3.2. Seal Strength of the Nanocomposites

Table 2 shows the average seal strength of PCPP film and the nanocomposites at 153 °C, 2.5 s and 350 kPa. The increase of the NS concentration generally tended to increase the seal strength of the PCPP. This fact could be associated with the ability of Si-O bonds of the NS to interconnect the polymeric chains through the silanol groups of NS and oxidized groups of PCPP during the heat sealing of the films, improving thermosealing ability. This improvement in the seal strength was also reported for starch films containing nano silicon dioxide [41]. Therefore, a higher NS concentration in the PCPP films favored an increase of heat transfer and strengthened its sealing. Furthermore, higher standard deviations in the seal strength values were obtained at higher NS concentrations attributed to a greater heterogeneous dispersion of the filler within the film (see Figure 1). Nonetheless, 0.5 and 1 wt% of NS1 produced the lowest seal strength of the PCPP. This fact could be associated with the hydrophilic nature of NS1, promoting low miscibility between the PCPP and the nanoparticles better dispersed at low concentrations, as verified by SEM analysis (Figure 1). A better dispersion increased the interfacial polymer-nanosilica area that resulted from the thermosealing, and thus, promoted the presence of a higher number of weak adhesion points that could be triggered by seal failure. In contrast, the hydrophobic NS2 could favor the miscibility with the polymer, and thus, a greater seal strength was reached. The better miscibility between organically modified NS and several polymers have been evidenced in previous works. Hahm et al. (2003) observed that the replacement of silanol groups of the NS by trimethylsilyl groups favored better miscibility with PET and dispersion in the nanocomposite [42].

Regarding the type of failure, all nanocomposites showed an adhesive peel failure, except for the sample PCPP-4NS1 which showed 2/6 cases of material break failures (Table 2). This latter can be attributed to the lower ductility of the film PCPP-4NS1, as was reported in the mechanical analysis, which increased the possibility of breakage of film in the seal area when stress was applied instead of the seal peeling. 

### 3.4. Barrier Properties: WVP and OP

The WVP and OP of the nanocomposites at 1 wt% of NS are shown in Table 3. In polymeric matrices, permeability is a phenomenon that occurs after permeant molecules are sorbed and diffused through the matrix. In plastic materials, the permeability depends on the combination and predominance of some of the following parameters: type of polymer, chain stiffness, chain entanglements and interactions, film thickness, free volume, crystallinity, presence of fillers, type of fillers, as well as external factors (humidity, temperature) [43].

As Table 3 shows, the WVP of the VPP was 1.8-fold lower than PCPP. This fact would be associated to the low crystallinity of the PCPP (Figure S2, Supplementary Materials) and its lower hydrophobicity due to the presence of oxidized groups formed during the thermo-oxidative processes in the recycling which was verified by ATR-FTIR analysis (Figure S1, Supplementary Materials). Furthermore, the presence of PE in the post-consumer plastic could favor the increment of WVP since this polymer exhibits a poor barrier property to water vapor [44]. Meanwhile, the incorporation of NS in the PCPP did not produce significant changes in the WVP of the polymer. This result evidenced that the nanoparticles were well dispersed in the polymeric matrix when incorporated at 1 wt% even when NS1 was used.

Regarding the oxygen permeability, interestingly, PCPP film exhibited an OP 6-fold lower than those obtained for VPP film. Although PCPP maintained a lower crystallinity, the diffusion of the oxygen molecules was impeded by more tortuosity in the polymeric matrix which was the predominant effect. The higher tortuosity in PCPP occurred as a result of higher intensive interactions between polymeric chains due to a higher number of oxidized groups and other additives in the recycled polymer compared to the VPP. On the other hand, like WVP, the nanocomposites did not show significant changes in the OP of the PCPP, and this fact could be newly associated with a good dispersion of NS in the polymeric matrix. 

### 3.5. Overall Migration (OM)

OM was determined in order to evaluate the safety of the packaging material to be in contact with fatty food. OM from VPP and PCPP films as well as nanocomposites with both NS at 1 wt% and 4 wt% are shown in Table 4. The results demonstrated that the OM values of compounds from VPP were statistically lowest compared to PCPP and the nanocomposite films, being lower than the limit of OM of 10 mg dm^−2^ established by the EU Standard N° 10/2011. This value is equivalent to 60 mg per kg of food occupying a volume of a cubic package with 6 dm^2^ of total area. The OM of the PCPP, which surpassed the limit of 10 mg dm^−2^, could be associated with the migration of oligomers and other substances of low molar mass resulting from the polymer degradation mainly occurring during the thermo-mechanical cycles of the recycling process. A previous study based on blends of virgin and recycled PET also associated the high values of overall migration with the presence of polymer degraded by recycling [45]. In addition to this effect, the great affinity between the PCPP and the fatty simulant could also facilitate the migration of inorganic particles and additives such as slip, anti-static and nucleating agents as well as rheology modifiers incorporated during the preparation of the plastic materials [46]. 

Conversely, statistical differences were not found between OM of the PCPP and nanocomposites. Nonetheless, it was observed that the incorporation of NS at the studied concentrations in the PCPP produced a slight tendency to decrease OM until reaching a value similar for all nanocomposite films. This decrease could be associated with a tortuous path in the polymeric matrix due to NS producing a barrier effect to the migration of compounds from the nanocomposites towards the simulant. This phenomenon was also evidenced in our previous studies on migration from nanocomposites based on nanoclays. In both cases, incorporating nanoclays in post-consumer recycled PP and PET films diminished OM to fatty simulants [7,16]. 

## 4. Conclusions

The nanocomposite at 1 wt% of fumed silica NS showed the best mechanical performance under the studied conditions. The better dispersion and interaction of both nanofillers, hydrophilic and hydrophobic, at low NS concentration with the polymeric matrix, and good interfacial adhesion, favored an increase of Young’s modulus and tensile strength of the PCPP. However, the presence of some agglomerations of NS at higher NS content favored premature breakage of the film. It is noteworthy that NS addition promoted the heat-sealing ability of the PCPP, tending to higher seal strength values when NS loading increased, more significantly when organically modified NS was used due to the better affinity between PCPP and nanoparticles which caused stronger adhesion points. Regarding the water and oxygen permeability of the PCPP, these were not affected by the presence of the NS. Interestingly, the presence of oxidized groups in the recycled plastic resulted in a greater tortuosity for the oxygen path.

On the other hand, the overall migration from the control PCPP and nanocomposites films with 1 wt% and 4 wt% of NS to the fatty simulant overcame the limit of 10 mg dm^−2^ established by the EU Standard N° 10/2011. Nonetheless, hydrophilic and hydrophobic fumed silicas exerted a barrier effect and reduced the migration of compounds from the nanocomposite films toward the fatty simulant. Thus, NS enhanced the performance of the PCPP for being used in food packaging applications but further research and innovation for reducing overall migration to a fatty simulant are required.

## Figures and Tables

**Table 1:** Tensile parameters of control and nanocomposite films.

Film	YM (MPa)	TS (MPa)	EB (%)	Thickness (µm)
VPP	1163 ± 61 ^f^	39.0 ± 0.7 ^e^	21.2 ± 6.0 ^cd^	162 ± 13
PCPP	608 ± 65 ^bcde^	19.8 ± 0.9 ^b^	23.7 ± 8.2 ^d^	148 ± 10
PCPP-0.5 NS1	569 ± 63 ^abc^	20.2 ± 0.7 ^bc^	14.3 ± 3.4 ^b^	159 ± 9
PCPP-1NS1	657 ± 80 ^de^	21.8 ± 1.1 ^d^	13.6 ± 1.8 ^b^	173 ± 10
PCPP-2NS1	547 ± 45 ^ab^	19.1 ± 1.4 ^b^	13.6 ± 3.5 ^b^	181 ± 7
PCPP-4NS1	537 ± 50 ^a^	17.2 ± 1.7 ^a^	9.2 ± 1.4 ^a^	181 ± 14
PCPP-0.5NS2	593 ± 62 ^abcd^	21.2 ± 2.0 ^cd^	15.1 ± 4.4 ^b^	164 ± 14
PCPP-1NS2	660 ± 70 ^e^	21.6 ± 1.6 ^d^	13.7 ± 3.5 ^b^	177 ± 14
PCPP-2NS2	602 ± 111 ^bcde^	20.0 ± 2.2 ^bc^	15.4 ± 3.4 ^b^	169 ± 7
PCPP-4NS2	612 ± 87 ^cde^	19.0 ± 1.6 ^b^	17.3 ± 4.5 ^bc^	164 ± 7

Superscripts a–f indicate significant differences in the parameters among samples according to the ANOVA analysis and LSD Fischer’s test (*p* < 0.05).

**Table 2:** Seal strength of the nanocomposites.

Film	Seal Strength (N m^−1^)	Type of Failure
PCPP	108.3 ± 88.4 ^ab^	AP
PCPP-0.5NS1	25.5 ± 8.3 ^a^	AP
PCPP-1NS1	39.1 ± 13.9 ^a^	AP
PCPP-2NS1	154.4 ± 39.4 ^b^	AP
PCPP-4NS1	188.3 ± 140.6 ^b^	AP (4/6), MB (2/6)
PCPP-0.5NS2	127.5 ± 82.0 ^ab^	AP
PCPP-1NS2	115.8 ± 47.2 ^ab^	AP
PCPP-2NS2	168.0 ± 126.0 ^b^	AP
PCPP-4NS2	196.8 ± 145.3 ^ab^	AP

Superscripts a and b indicate significant differences among the samples according to the ANOVA analysis and LSD Fischer’s test (*p* < 0.05). Average thicknesses of the films: 150 to 180 µm. AP: adhesive pealing. MB: material break. Six replicates were tested for each sample.

**Table 3:** Water vapor and oxygen permeability of the films.

Film	WVP × 10^5^ (g mm m^−2^ d Pa^−1^)	OP (cm^3^ mm m^−2^ day^−1^ atm^−1^)
VPP	3.49 ± 0.05 ^a^	453.93 ± 115.14 ^b^
PCPP	6.47 ± 0.26 ^b^	80.75 ± 8.52 ^a^
PCPP-1NS1	6.48 ± 0.20 ^b^	91.53 ± 10.87 ^a^
PCPP-1NS2	6.79 ± 0.57 ^b^	82.35 ± 6.77 ^a^

Superscripts a and b indicate significant differences among the samples according to the ANOVA analysis and LSD Fischer’s test (*p* < 0.05).

**Table 4:** Overall migration of VPP, PCPP and nanocomposite films to olive oil.

Film	Overall Migration (mg dm^−2^)
VPP	6.7 ± 1.4 ^a^
PCPP	17.3 ± 0.7 ^c^
PCPP-1NS1	15.6 ± 0.7 ^bc^
PCPP-4NS1	15.9 ± 0.1 ^bc^
PCPP-1NS2	15.3 ± 0.1 ^b^
PCPP-4NS2	15.9 ± 0.1 ^bc^

Superscripts a–c indicate significant differences among the samples according to the ANOVA analysis and LSD Fischer’s test (*p* < 0.05).

## Data Availability

Not applicable.

## Supplementary Materials

The following supporting information can be downloaded at: https://www.mdpi.com/article/10.3390/polym15051081/s1, Figure S1: ATR-FTIR spectra of VPP and PCPP films; Figure S2: DSC thermograms of VPP and PCPP films obtained during first heating.
