# Supplementary material for: Feasibility of Valorization of Post-Consumer Recycled Flexible Polypropylene by Adding Fumed Nanosilica for Its Potential Use in Food Packaging toward Sustainability
Source: Polymers (Basel). 2023 Feb 21;15(5):1081. doi: 10.3390/polym15051081 (PMC10005770; doi:10.3390/polym15051081)
Supplement: Supplementary file 1 [file polymers-15-01081-s001.zip › polymers-2217157-supplementary.pdf]

polymers

MDPI

Article

Feasibility of Valorization of Post-Consumer Recycled Flexible Polypropylene by Adding Fumed Nanosilica for Its Potential Use in Food Packaging toward Sustainability

Eliezer Velásquez [1,2,*], Carol López de Dicastillo [3], Cristian Patiño Vidal [1,2], Guillermo Copello [4,5], Cristopher Reyes [1], Abel Guarda [1,2,6] and María José Galotto [1,2,6]

1 Packaging Innovation Center (LABEN-Chile), University of Santiago of Chile (USACH), Santiago 9170201, Chile
2 Center for the Development of Nanoscience and Nanotechnology (CEDENNA), University of Santiago of Chile, Santiago 9170124, Chile
3 Packaging Laboratory, Institute of Agrochemistry and Food Technology (IATA-CSIC), 46980 Paterna, Spain
4 Universidad de Buenos Aires, Facultad de Farmacia y Bioquímica, Departamento de Ciencias Químicas, Junín 956, Buenos Aires C1113AAD, Argentina
5 CONICET—Universidad de Buenos Aires, Instituto de Química y Metabolismo del Fármaco (IQUIMEFA), Junín 956, Buenos Aires C1113AAD, Argentina
6 Food Science and Technology Department, Technological Faculty, University of Santiago of Chile (USACH), Santiago 9170201, Chile
* Correspondence: eliezer.velasquez@usach.cl

Abstract: The food industry has a current challenge of increasing the recycling of post-consumer plastics to reduce plastic waste towards a circular economy, especially flexible polypropylene, which is highly demanded in food packaging. However, recycling post-consumer plastics is limited because service life and reprocessing degrade their physical-mechanical properties and modify the migration of components from the recycled material to the food. This research evaluated the feasibility of valorization of post-consumer recycled flexible polypropylene (PCPP) by incorporating fumed nanosilica (NS). For this purpose, the effect of concentration and type (hydrophilic and hydrophobic) of NS on the morphological, mechanical, sealing, barrier and overall migration properties of PCPP films was studied. Incorporating NS improved Young's modulus and, more significantly, tensile strength at 0.5 wt% and 1 wt%, where a better particle dispersion was confirmed by EDS-SEM, but it diminished elongation at breakage of the films. Interestingly, NS tended to increase the seal strength of PCPP nanocomposite films more significantly at higher NS content, showing a seal failure of the adhesive peel type which is preferred for flexible packaging. NS at 1 wt% did not affect the water vapor and oxygen permeabilities of the films. Overall migration of PCPP and nanocomposites exceeded the limit value of 10 mg dm^{-2} allowed by European legislation at the studied concentrations of 1% and 4 wt%. Nonetheless, NS reduced the overall migration of PCPP from 17.3 to 15 mg dm^{-2} in all nanocomposites. In conclusion, PCPP with 1 wt% of hydrophobic NS presented an improved overall performance of the studied packaging properties.

Keywords: post-consumer polypropylene; fumed silica; food packaging; recycling

Supplementary material

This section contains information about ATR-FTIR and DSC analysis that support the findings detailed in the main manuscript.

A. Methods

A.1 Attenuated Total Reflectance-Fourier Transform Infrared Spectroscopy (ATR-FTIR)

The presence of oxidized groups and PE in the post-consumer recycled polypropylene were identified through FTIR equipment Bruker Alpha IFS 66V (Ettilingen, Germany) coupled to a crystal diamond of attenuated total reflection Bruker Platinum. FTIR spectra were obtained in Attenuated Total Reflectance (ATR) mode in a wavenumber range from 4000 to 400 cm^{-1} with a resolution of 4 cm^{-1} and 64 scans. The spectra analyses were performed with the program Opus v. 7.0

A.2 Differential scanning calorimetry (DSC)

Homopolymer VPP and PCPP films were analyzed through differential scanning calorimetry (DSC) using a Mettler DSC-822e analyzer (Schwarzenbach, Switzerland). 5 to 6 mg of each film were weighed into aluminum capsules and subjected to heating from 0 °C to 250 °C with a heating rate at 10 °C min^{-1} under a nitrogen atmosphere. Melting temperature (T_m) and melting enthalpy (ΔH_m) were determined. Furthermore, the crystallinity of the samples was calculated through equation 1:

$$X_c = \Delta H_m / (\Delta H_{100} * X_{PP}) \qquad \text{Equation 1}$$

Where ΔH_{100} is the melting enthalpy of a whole crystalline polypropylene (207 J g^{-1}) [1], and X_{PP} is the mass fraction of the polymer in the sample. DSC analyses were carried out in duplicate.

B. Results

B.1 Attenuated Total Reflectance-Fourier Transform Infrared Spectroscopy (ATR-FTIR)

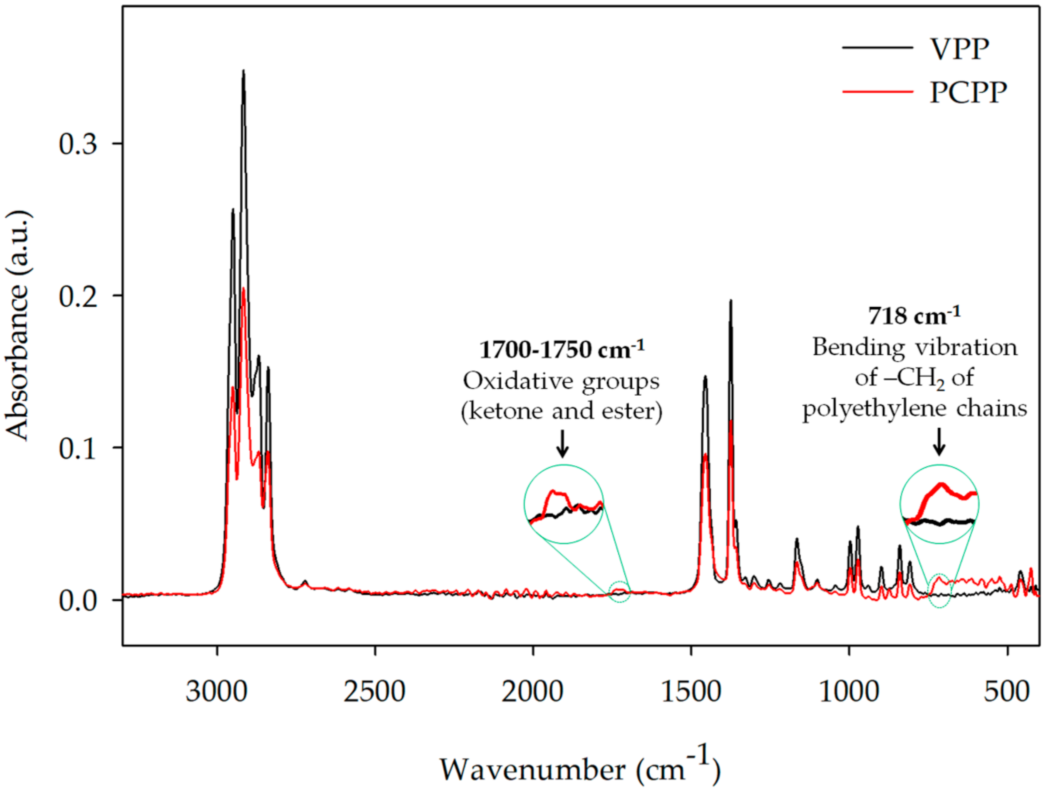

Figure S1. ATR-FTIR spectra of a reference virgin homopolymer polypropylene (VPP) and post-consumer recycled polypropylene (PCPP) films.

B.2 DSC analysis

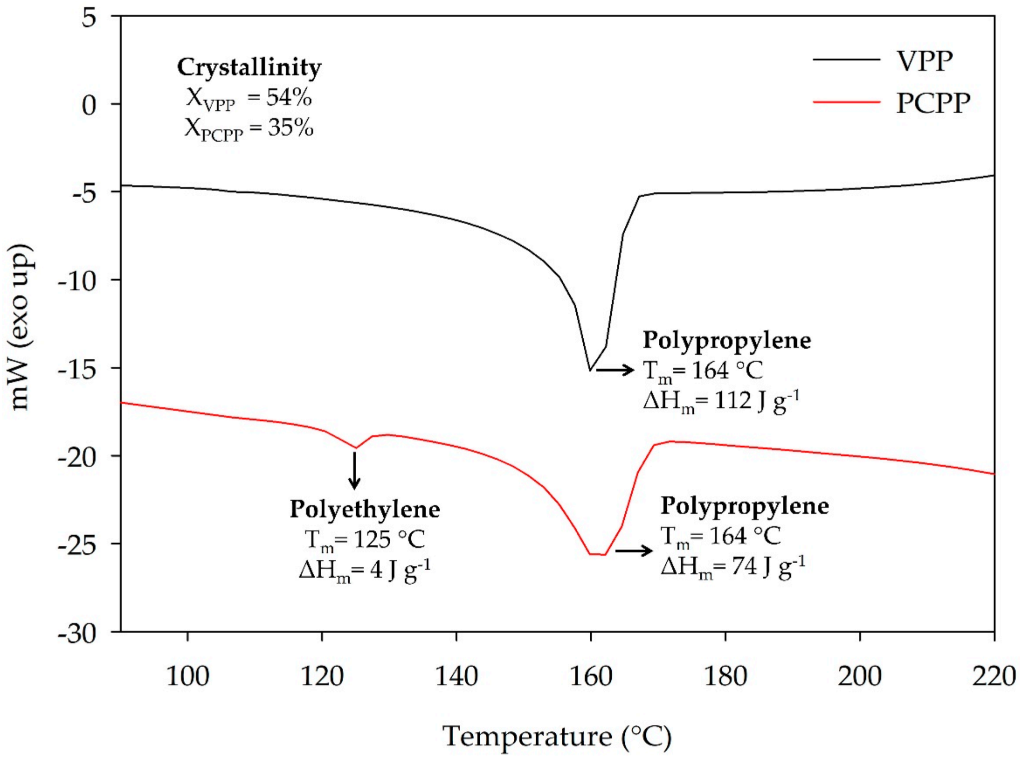

Figure S2. DSC first heating of reference virgin homopolymer polypropylene (VPP) and post-consumer recycled polypropylene (PCPP) films.
